# B chromosome dynamics in *Prochilodus costatus* (Teleostei, Characiformes) and comparisons with supernumerary chromosome system in other *Prochilodus* species

**DOI:** 10.3897/CompCytogen.v11i2.12784

**Published:** 2017-06-01

**Authors:** Silvana Melo, Ricardo Utsunomia, Manolo Penitente, Patrícia Elda Sobrinho-Scudeler, Fábio Porto-Foresti, Claudio Oliveira, Fausto Foresti, Jorge Abdala Dergam

**Affiliations:** 1 Departamento de Biologia Animal, Campus Universitário, Universidade Federal de Viçosa, 36570-000, Viçosa, Minas Gerais, Brazil; 2 Departamento de Morfologia, Instituto de Biociências, Universidade Estadual Paulista, Distrito de Rubião Junior, s/n, 18618-689, Botucatu, São Paulo, Brazil; 3 Departamento de Ciências Biológicas, Faculdade de Ciências, Universidade Estadual Paulista, Campus de Bauru, 17033-360 Bauru, São Paulo, Brazil

**Keywords:** additional chromosomes, genome, Prochilodontidae

## Abstract

Within the genus *Prochilodus* Agassiz, 1829, five species are known to carry B chromosomes, i.e. chromosomes beyond the usual diploid number that have been traditionally considered as accessory for the genome. Chromosome microdissection and mapping of repetitive DNA sequences are effective tools to assess the DNA content and allow a better understanding about the origin and composition of these elements in an array of species. In this study, a novel characterization of B chromosomes in *Prochilodus costatus* Valenciennes, 1850 (2n=54) was reported for the first time and their sequence complementarity with the supernumerary chromosomes observed in *Prochilodus lineatus* (Valenciennes, 1836) and *Prochilodus argenteus* Agassiz, 1829 was investigated. The hybridization patterns obtained with chromosome painting using the micro B probe of *P. costatus* and the satDNA SATH1 mapping made it possible to assume homology of sequences between the B chromosomes of these congeneric species. Our results suggest that the origin of B chromosomes in the genus *Prochilodus* is a phylogenetically old event.

## Introduction

Supernumerary or B chromosomes are dispensable genomic elements found in approximately 15% of eukaryotes (Camacho et al. 2000). Usually, these elements are assumed to have derived from standard genomic elements (e.g. A or sex chromosomes) from the same (intraspecific origin) or a different (interspecific origin) species (reviewed in Camacho et al. 2000). As a result of their reduced recombination rates, these elements are prone to accumulate several types of repetitive DNA sequences during their evolution (Camacho 2005). For instance, ribosomal and histone clusters, snDNA genes and satellite DNAs have been extensively found on B chromosomes of several species and provided evidence for the origin of these elements in different organisms (Teruel et al. 2009, Silva et al. 2014, 2016, Menezes-de-Carvalho et al. 2015, Utsunomia et al. 2016).

*Prochilodus* is the most species-rich genus within family Prochilodontidae and its species exhibit a well-preserved karyotypic macrostructure, a diploid number of 54 chromosomes and karyotypic formula of 40m + 14sm (Pauls and Bertollo 1983, 1990, Venere et al. 1999, Oliveira et al. 2003). However, prominent intra- and interspecific differences have been reported regarding the frequency and occurrence of B chromosomes. To date, supernumerary elements were reported in five species: *Prochilodus lineatus* (Valenciennes, 1836), *Prochilodus brevis* Steindachner, 1874, *Prochilodus nigricans* Agassiz, 1829, *Prochilodus mariae* Eigenmann, 1922, and *Prochilodus argenteus* Agassiz, 1829 (Pauls and Bertollo 1983, 1990, Venere et al. 1999, Oliveira et al. 2003, Penitente et al. 2015); however, information regarding the origin, molecular content, and populational dynamics of these B chromosomes are restricted to *P. lineatus* (Maistro et al. 2000, Jesus et al. 2003, Artoni et al. 2006, Voltolin et al. 2010, 2013a, Penitente et al. 2013).

In a previous study, Jesus et al. (2003) isolated two satellite DNA families from the *P. lineatus* genome, SATH1 and SATH2, mainly located in the pericentromeric region of chromosomes. Remarkably, in this species the SATH1 satDNA was associated with both A and B chromosomes, suggesting an intra-specific origin of these elements (Artoni et al. 2006, Vicari et al. 2010). However, no information related to the chromosomal location of SATH1 in congeneric species is available, which could be useful in understanding the origin and dynamics of B chromosomes in this genus.

*Prochilodus costatus* Valenciennes, 1850 is an endemic species of the São Francisco River basin and previous cytogenetic analyses did not reveal the presence of B chromosomes in this species (Pauls and Bertollo 1990, Galetti 1991, Voltolin et al. 2013a, 2013b). In this study, we described the occurrence of B chromosomes in *P. costatus* for the first time and performed a comparative analysis with other B chromosome systems found in *P. lineatus* and *P. argenteus* using chromosome painting and mapping of SATH1 satDNA.

## Methods

*Prochilodus costatus* specimens (N=23) were collected in three distinct sites along São Francisco River basin (Table 1), Minas Gerais, Brazil, with SISBIO14975-1 permission. One sample of *Prochilodus argenteus* was collected near the Três Marias Dam, from the São Francisco River basin and one sample of *Prochilodus lineatus* was collected in Volta Grande Dam - MG, from the Grande River basin.

**Table 1.** B chromosome frequency and Mitotic Instability index (MI) of somatic cells in *P. costatus*.

Sample	Locality	Geographic coordinates	Number of B per cell		MB	N	MI
			*0B*	*1B*			
JD5480	São Francisco River – Iguatama	20°09'50"S, 45°43'08"W	12	–	0B	12	0
JD5481	São Francisco River – Iguatama	20°09'50"S, 45°43'08"W	29	1	0B	30	0.01
JD5482	São Francisco River – Iguatama	20°09'50"S, 45°43'08"W	28	2	0B	30	0.01
JD5483	São Francisco River – Iguatama	20°09'50"S, 45°43'08"W	–	30	1B	30	0
JD5486	São Francisco River – Iguatama	20°09'50"S, 45°43'08"W	15	–	0B	15	0
JD5490	São Francisco River – Iguatama	20°09'50"S, 45°43'08"W	27	3	0B	30	0.01
JD5497	São Francisco River – Iguatama	20°09'50"S, 45°43'08"W	29	1	0B	30	0.01
JD5502	São Francisco River – Iguatama	20°09'50"S, 45°43'08"W	17	1	0B	18	0.11
JD5604	São Francisco River – Iguatama	20°09'50"S, 45°43'08"W	27	3	0B	30	0.01
JD5605	São Francisco River – Iguatama	20°09'50"S, 45°43'08"W	27	3	0B	30	0.01
JD5619	São Francisco River – Iguatama	20°09'50"S, 45°43'08"W	30	–	0B	30	0
JD5620	São Francisco River – Iguatama	20°09'50"S, 45°43'08"W	16	1	0B	17	0.01
JD5517	Pandeiros River – Januária	15°23'28"S, 44°53'37"W	30	–	0B	30	0
JD5531	Pandeiros River – Januária	15°23'28"S, 44°53'37"W	27	3	0B	30	0.01
JD5562	São Francisco River – Três Marias Dam	18°08'47"S, 45°13'37"W	25	5	0B	30	0.1
JD5563	São Francisco River – Três Marias Dam	18°08'47"S, 45°13'37"W	11	–	0B	11	0
JD5565	São Francisco River – Três Marias Dam	18°08'47"S, 45°13'37"W	15		0B	15	0
JD5566	São Francisco River – Três Marias Dam	18°08'47"S, 45°13'37"W	15	1	0B	16	0.04
CT4639	São Francisco River – Três Marias Dam	18°08'47"S, 45°13'37"W	21	6	0B	27	0.01
CT4640	São Francisco River – Três Marias Dam	18°08'47"S, 45°13'37"W	23	1	0B	24	0.04
CT4644	São Francisco River – Três Marias Dam	18°08'47"S, 45°13'37"W	10	–	0B	10	0
CT4645	São Francisco River – Três Marias Dam	18°08'47"S, 45°13'37"W	10	–	0B	10	0
CT4647	São Francisco River – Três Marias Dam	18°08'47"S, 45°13'37"W	10	–	0B	10	0
							X_MI_=0,0292

MB: modal number of B chromosomes; N: number of metaphases analyzed; MI: Mitotic Instability index; X_MI_: MI average among individuals with B chromosomes.

Before analysis, the animals were anesthetized and euthanized with a 300 mg L^-1^ clove oil aqueous solution (Lucena et al. 2013) in accordance with the Universidade Federal de Viçosa Animal Welfare Committee authorization #35/2014. The mitotic chromosomes were obtained from cell suspensions from the anterior kidney (Bertollo et al. 1978) and C-banding technique was carried out according to Sumner (1972). The chromosomes were classified as metacentric (m), submetacentric (sm), subtelocentric (st) and acrocentric (a), modified from Levan et al. (1964).

Because the B chromosome frequency was variable among cells within the same individual, an analysis of mitotic instability causing this variation was performed. For this purpose, we used a mitotic instability index previously developed in a migratory locust (Pardo et al. 1995) that is based on the assumption that the median number of B chromosomes in the adult represents the number of B chromosomes in the zygotic stage. This mitotic instability index (MI) estimates the sum of deviations in B numbers in a sample of cells with respect to the median, normalized per B chromosome.

Microdissection was performed in an Eppendorf TransferMan NK2 micromanipulator attached to a Zeiss Axiovert 100 microscope. Ten B chromosomes were microdissected from the same *P. costatus* specimen (JD5483) carrying one B chromosome. The microdissected DNAs were placed in 9 μl of DNase-free ultrapure water and then fragmented and amplified using the GenomePlex Single Cell Whole Genome Amplification Kit (wga4-Sigma) (Gribble et al. 2004). After the initial amplification, we obtained a B chromosome DNA probe (BPC probe) labeled with digoxigenin-11-dUTP (Roche Applied Science) using the GenomePlex Whole Genome Amplification Reamplification Kit (wga3-Sigma), following the manufacturer’s protocol. This BPC probe was hybridized on metaphase plates of *P. costatus*, *P. lineatus* and *P. argenteus* .

Considering that SATH1 satDNA was described to occur on the B chromosomes of *P. lineatus* (Jesus et al. 2003, Artoni et al. 2006), the distribution of this probe in different *Prochilodus* species would be relevant for understanding evolutionary aspects of this B chromosome system. Thus, three SATH1 sequences (AF363731.1, AF363732.1 and AF363734.1) were retrieved from GenBank and aligned using MUSCLE algorithm (Edgar 2004). Subsequently, the convergent primers SATH1-F 5’-GCTGCAGCAAAAACCCTACC- 3’ and SATH1-R 5’-AGTGGGAGCTAGGGTTAGGG-3’ were designed on conserved regions to yield a 563bp PCR product, suitable for FISH (Suppl. material 1). The reactions were performed in 1x PCR buffer, 1.5 mM MgCl_2_, 200 μM each dNTP, 0.5 U of *Taq* polymerase (Invitrogen), 0.1 μM each primer and 5 ng of DNA. The PCR products were checked in 2% agarose gels (Suppl. material 2). After amplification, PCR products were labeled with digoxigenin-11-dUTP.

Fluorescent *in situ* hybridization (FISH) was performed under high stringency conditions using the method described by Pinkel et al. (1986) with modifications. Pre-hybridization: slides were incubated with 0,005% pepsin/10mM HCl for 10 min at 37 °C and the chromosomal DNA was denatured in 70% formamide/2xSSC for 5 min at 70 °C. For each slide, 30 μl of hybridization solution (containing 200 ng of labeled probe, 50% formamide, 2xSSC and 10% dextran sulphate) was denatured for 10 min at 95 °C, then dropped onto the slides and allowed to hybridize overnight at 37 °C in a moist chamber. Post hybridization: slides were washed in 0,2xSSC/15% formamide for 20 min at 42 °C, followed by a second wash in 0,1xSSC for 15 min at 60 °C and a final wash at room temperature in 4xSSC/0,5% Tween for 10 min. Probe detection was carried out with anti-digoxigenin-rhodamine (Roche), and the chromosomes were counterstained with DAPI (4’,6-diamidino-2-phenylindole, Vector Laboratories) and analyzed using an optical photomicroscope (Olympus BX61). Images were captured with an Olympus DP70 digital camera using the Image Pro plus 6.0 software (Media Cybernetics). From each individual, a minimum of five cells was analyzed for FISH.

## Results and discussion

**Figure 1.**
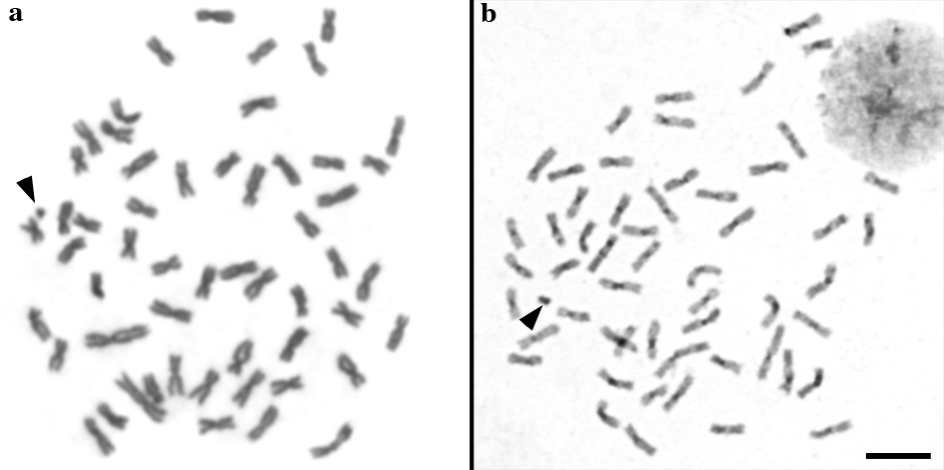
Metaphases of *P. costatus* with conventional staining (**a**) and C-banding (**b**). Arrowheads indicate the supernumerary chromosomes. Bar = 5 μm.

The analyzed samples of *P. costatus* showed the expected karyotypic macrostructure of 54 chromosomes (40m + 14sm) (Fig. 1), a conserved trait among Prochilodontidae, indicating that large chromosome rearrangements are apparently unusual in this fish group (Feldberg et al. 1987, Pauls and Bertollo 1983, 1990, Venere et al. 1999, Oliveira et al. 2003, Voltolin et al. 2013, Penitente et al. 2015).

Mitotically unstable B chromosomes were observed in 14 of 23 *P. costatus* specimens analyzed, ranging from 0 to 1 B chromosome per cell, characterizing the sixth species within *Prochilodus* carrying these elements (Table 1). Although chromosome numbers and karyotype structure are highly stable in *Prochilodus* species, the frequency of different B chromosomes in distinct species/populations is remarkable (Cavallaro et al. 2000, Artoni et al. 2006, Penitente et al. 2015). For instance, two populations of *P. costatus* were analyzed until now and none of them showed B chromosomes in their cells (Pauls and Bertollo 1990, Voltolin et al. 2013a, 2013b). In fact, supernumerary chromosomes in fish are usually highly dynamic elements, and the existence of B-lacking and B-carrying populations is quite common and largely known (Oliveira et al. 2009).

The Mitotic Instability calculation resulted in an average index of 0.0292 (Table 1). This MI index is considered low and indicates that this elements may be testifying a process of stabilization on *P. costatus* populations, as suggested for populations of *P. lineatus* (Cavallaro et al. 2000) and *P. argenteus* (Penitente et al. 2015).

C-banding revealed small pericentromeric heterochromatic regions in all chromosomes (Fig. 1b), with a narrow band in the metacentric chromosome pair number 2, as reported for other species of *Prochilodus*, except in *P. lineatus* (Artoni et al. 2006, Vicari et al. 2006, Voltolin et al. 2013a). Additionally, the supernumerary chromosomes were entirely C-band positive (Fig. 1b; arrowhead).

**Figure 2.**
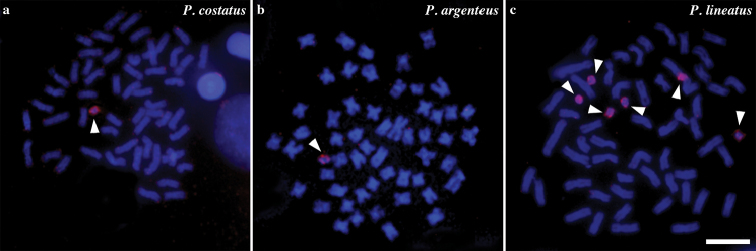
Chromosome painting with BPC probe on metaphases of *P. costatus* (**a**), *P. argenteus* (**b**) and *P. lineatus*. Arrowheads indicate the supernumerary chromosomes. Bar = 10 μm.

Cross-species chromosome painting showed that the BPC probe hybridized on the B chromosomes of *P. costatus*, *P. lineatus*, and *P. argenteus* (Fig. 2). Notably, this hybridization pattern evidenced that all B chromosomes analyzed in this study shared anonymous sequences, as already reported for *P. lineatus* and *P. nigricans* (Voltolin et al. 2013b). The identical hybridization pattern generated throughout chromosome painting between different species or populations allow us to suggest that these B chromosomes show a high degree of homology. Notably, such hypothesis should be better tested in the future since chromosome painting is not a conclusive method when studying B chromosomes origin in closely related species (Silva et al. 2016). However, one must say that the association of this technique with known repetitive sequences mapping by FISH may provide additional information about the DNA content and sequence homology in supernumerary chromosomes of different species.

**Figure 3.**
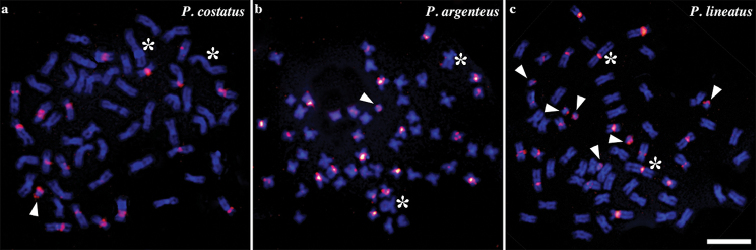
Metaphases of *P. costatus* (**a**), *P. argenteus* (**b**) and *P. lineatus* hybridized with SATH1 probe. The asterisks indicate the first pair of metacentric chromosomes and the arrowheads indicate the supernumerary chromosomes. Bar = 10 μm.

FISH experiments revealed large clusters of SATH1 satDNA in the pericentromeric regions of many A-chromosomes in the three studied species. Remarkably, the largest metacentric chromosome of *P. lineatus* exhibited a strong signal in the pericentromeric region, differently from *P. costatus* and *P. argenteus* (Fig. 3; asterisks), characterizing an interesting chromosomal marker. In addition, our results evidenced that the supernumerary chromosomes of *P. costatus* (one B chromosome), *P. argenteus* (one B chromosome) and *P. lineatus* (six B chromosomes) carry the SATH1 satDNA sequences (Fig. 3; arrowheads). Notably, SATH1 sequences were also extensively spread over several A chromosomes in all species, whereas the BPC and all previously microdissected B-probes of *Prochilodus* (Voltolin et al. 2013b) revealed signals only on the B chromosomes. Such deviation might be caused by a possible bias of amplification in the GenomePlex reaction. Thus, several different repetitive DNA elements might be located on these B chromosomes and deserve further investigation.

Cytogenetic data show a conservative trend within the family Prochilodontidae, with a diploid number of 2n=54 biarmed chromosomes (Pauls and Bertollo 1983, 1990, Feldberg et al. 1987, Voltolin et al. 2013, Nirchio-Tursellino et al. 2016). However, *Semaprochilodus* Fowler, 1941 and *Prochilodus* genera exhibit the presence of sex related and supernumerary chromosomes, respectively, different from *Ichthyoelephas* Posada Arango, 1909, in which these elements are absent. Accordingly, recent molecular phylogenetic analyses (Melo et al. 2016) proposed the monophily of Prochilodontidae and placed *Ichthyoelephas* as a sister group of the *Semaprochilodus* + *Prochilodus* clade. The absence of sex related and supernumerary chromosomes, together with differences on the location of the repetitive 5S and 18S rDNA, suggest a plesiomorphic position of the *Ichthyoelephas* karyotype (Nirchio-Tursellino et al. 2016), involving at least two rearrangements events in the common ancestor of the Prochilodontidae.

Only eight out of the 13 valid species of *Prochilodus* have been karyotyped and the presence of B chromosomes was reported for six species. In this sense, B chromosomes are present in most species, except for the *P. vimboides* and the trans-Andean clade. However, our results suggest that these chromosomes may be absent in some populations, or their low frequency may demand higher sampling efforts. The hybridization patterns of both SATH1 satDNA and chromosome painting with the B-specific probe suggested an old and intraspecific origin of B chromosomes within this genus.

## Supplementary materials

Supplementary material 1 Alignment of three SATH1 satDNA sequences from *Prochilodus lineatus* genome Data type: molecular data Explanation note: Alignment of three SATH1 satDNA sequences from *Prochilodus lineatus* genome (accession no. AF363731.1, AF363732.1 and AF363734.1). Designed primers are boxed, evidencing a 563bp amplicon size. This dataset is made available under the Open Database License (http://opendatacommons.org/licenses/odbl/1.0/). The Open Database License (ODbL) is a license agreement intended to allow users to freely share, modify, and use this Dataset while maintaining this same freedom for others, provided that the original source and author(s) are credited.Silvana Melo, Ricardo Utsunomia, Manolo Penitente, Patrícia Elda Sobrinho-Scudeler, Fábio Porto-Foresti, Claudio Oliveira, Fausto Foresti, Jorge Abdala Dergam

Supplementary material 2 B chromosome microdissection process and SATH1 satDNA amplification checked in 2% agarose gel Data type: molecular data Explanation note: Amplification patterns of the B chromosome after WGA (B-probe lane) and the SATH1 satDNA (SATH1 lane) from *P. costatus* genome. L: 1 Kb Plus DNA Ladder. This dataset is made available under the Open Database License (http://opendatacommons.org/licenses/odbl/1.0/). The Open Database License (ODbL) is a license agreement intended to allow users to freely share, modify, and use this Dataset while maintaining this same freedom for others, provided that the original source and author(s) are credited.Silvana Melo, Ricardo Utsunomia, Manolo Penitente, Patrícia Elda Sobrinho-Scudeler, Fábio Porto-Foresti, Claudio Oliveira, Fausto Foresti, Jorge Abdala Dergam
