# Supplementary material for: B chromosome dynamics in Prochilodus costatus (Teleostei, Characiformes) and comparisons with supernumerary chromosome system in other Prochilodus species
Source: Comp Cytogenet. 2017 Jun 1;11(2):393–403. doi: 10.3897/CompCytogen.v11i2.12784 (PMC5596993; doi:10.3897/CompCytogen.v11i2.12784)
Supplement: Supplementary material 1 — Alignment of three SATH1 satDNA sequences from Prochilodus lineatus genome [file comparative_cytogenetics-11-393-s001.pdf]

```
                                    10        20        30        40        50        60        70        80        90       100
                            ....|....|....|....|....|....|....|....|....|....|....|....|....|....|....|....|....|....|....|....|
gi|14039759|gb|AF363732.1|  AGCTTAACTGGAGTTATCAGGCTGGCTACAGCACTAACTCTTGATTAAGCCATACAATTACCTGGAGCAACTAACCTTGCTCCAGCACTAATGCAAGTTA
gi|14039762|gb|AF363734.1|  ............................A.......................................................................
gi|14039757|gb|AF363731.1|  ........................C.................................................A.....-...................

                                   110       120       130       140       150       160       170       180       190       200
                            ....|....|....|....|....|....|....|....|....|....|....|....|....|....|....|....|....|....|....|....|
gi|14039759|gb|AF363732.1|  CAACTCTAACCCTAGCTCCCACTTCATATCTAGCCCAAGCGCTAAGTCTAGCTGTCACTTAACCCTAGTTGGAGACATCAACTTAACTCCAGTACTCATT
gi|14039762|gb|AF363734.1|  ............................................................................T...............C.......
gi|14039757|gb|AF363731.1|  ...........A................................................................T...................A...

                                   210       220       230       240       250       260       270       280       290       300
                            ....|....|....|....|....|....|....|....|....|....|....|....|....|....|....|....|....|....|....|....|
gi|14039759|gb|AF363732.1|  CTAGCTATAGCACTAACTTTTGATTAAGCCCTAAAAGTAGCTGCAGCAAAAACCCTACCCTTAGCACTAACCTGA-GGCTCAACCCTAACCCTAGCTGGA
gi|14039762|gb|AF363734.1|  ........................................................................C..-........................
gi|14039757|gb|AF363731.1|  ........................................................................C..-......T.................

                                   310       320       330       340       350       360       370       380       390       400
                            ....|....|....|....|....|....|....|....|....|....|....|....|....|....|....|....|....|....|....|....|
gi|14039759|gb|AF363732.1|  -CCATTAAGCCTAGTTCAAGCGCTAAACCCAGCTGAAACCCATACTCTCGTTCCAGTCCTAAAATTAACTCCAGACTTCATGCTTGCTACAACAATAAAA
gi|14039762|gb|AF363734.1|  -.....................T............G.T....-..........A..............................A.........C...TT
gi|14039757|gb|AF363731.1|  -..................................G...G..A..........A..............................A.........C...TT

                                   410       420       430       440       450       460       470       480       490       500
                            ....|....|....|....|....|....|....|....|....|....|....|....|....|....|....|....|....|....|....|....|
gi|14039759|gb|AF363732.1|  CTTGATTAAGCCTTAAAATGTCCTGCAGCAATAAACCTTCCTCCAGCACTAACGTGAGCAGCAGCCTTAACCCTAGCTCCCAGCCCAACTCAAGCTCAGG
gi|14039762|gb|AF363734.1|  ......................................GN.......N...TA.....G...N.T......................A...........
gi|14039757|gb|AF363731.1|  A....................................G.G...................G...A......G.......T.........A...........

                                   510       520       530       540       550       560       570       580       590       600
                            ....|....|....|....|....|....|....|....|....|....|....|....|....|....|....|....|....|....|....|....|
gi|14039759|gb|AF363732.1|  CAATAACCATGGCTGAAAGACAAACCCTTGGTTTAATCTTAGTTCTTAACTACAGNAAACATGCTAGCTAGAGCAC-TAACTCTGAAGTATATCATAAAC
gi|14039762|gb|AF363734.1|  .C.A..........................T..C.......A..................................-....................C..
gi|14039757|gb|AF363731.1|  .C...............G...............C.......A.....-.............G..............T.....A..............C..

                                   610       620       630       640       650       660       670       680       690       700
                            ....|....|....|....|....|....|....|....|....|....|....|....|....|....|....|....|....|....|....|....|
gi|14039759|gb|AF363732.1|  TTAGCTACAGTAATATACCTTTGNGCAGATGTAAGACAAGCTGCAAGCCCAACCTTAGGTNNTACACCAAACCTNAATTNAGCGGTNACCCTAGCGGTTA
gi|14039762|gb|AF363734.1|  ..........C......................G........................C...A..C..................C...........C...
gi|14039757|gb|AF363731.1|  ..........C...............................................C...A..C..................C...A.......A...

                                   710       720       730       740       750       760       770       780       790       800
                            ....|....|....|....|....|....|....|....|....|....|....|....|....|....|....|....|....|....|....|....|
gi|14039759|gb|AF363732.1|  ATCAAACCCTCATTCAAGTTTTAAACTTAACTCCAGTCCTCATCCCTAGCCACAGCANTAACTTTTGATTAGGACCTTAAATTACCTGCAGCAATGAATT
gi|14039762|gb|AF363734.1|  C..................C.......................-.T......................A....C.T........................
gi|14039757|gb|AF363731.1|  C..C...............C.G.....................-.............................CAT........................

                                   810       820       830       840       850       860       870       880       890       900
                            ....|....|....|....|....|....|....|....|....|....|....|....|....|....|....|....|....|....|....|....|
gi|14039759|gb|AF363732.1|  TTTCTCTGGCTTAAAGGCAAGCTGCAACCCTAACCNTAGCTCCCANTCTAAATNTTGCTCAAGCGCTAACCATATCTGTAAATCAAACCCCCATTCAACT
gi|14039762|gb|AF363734.1|  ..........A..............................A.G........................................................
gi|14039757|gb|AF363731.1|  ..........A..............................................NG-GN......................................

                            ....
gi|14039759|gb|AF363732.1|  CTTA
gi|14039762|gb|AF363734.1|  ....
gi|14039757|gb|AF363731.1|  ....
```

Figure S1. Alignment of three SATH1 satDNA sequences from *Prochilodus lineatus* genome (accession no. AF363731.1, AF363732.1 and AF363734.1). Designed primers are boxed, evidencing a 563bp amplicon size.
