# Supplementary material for: B chromosome dynamics in Prochilodus costatus (Teleostei, Characiformes) and comparisons with supernumerary chromosome system in other Prochilodus species
Source: Comp Cytogenet. 2017 Jun 1;11(2):393–403. doi: 10.3897/CompCytogen.v11i2.12784 (PMC5596993; doi:10.3897/CompCytogen.v11i2.12784)
Supplement: Supplementary material 2 — B chromosome microdissection process and SATH1 satDNA amplification checked in 2% agarose gel [file comparative_cytogenetics-11-393-s002.pdf]

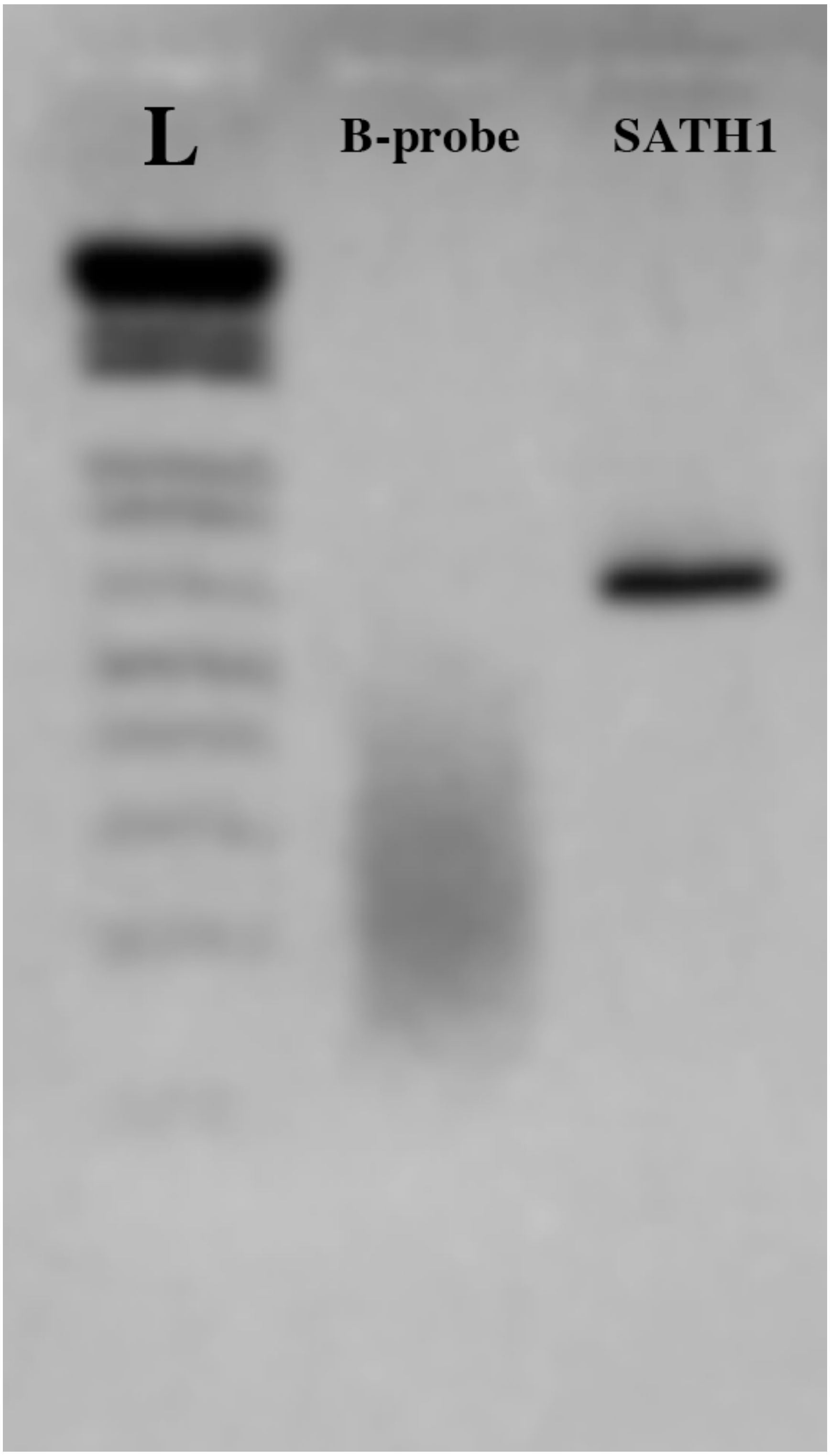

Figure S2. Amplification patterns of the B chromosome after WGA (B-probe lane) and the SATH1 satDNA (SATH1 lane) from *P. costatus* genome. L: 1 Kb Plus DNA Ladder.
